# Enhanced Absorption and Diffusion Properties of Lithium on B,N,V_C_-decorated Graphene

**DOI:** 10.1038/srep37911

**Published:** 2016-11-29

**Authors:** Mengting Jin, L. C. Yu, W. M. Shi, J. G. Deng, Y. N. Zhang

**Affiliations:** 1 Chengdu Green Energy and Green Manufacturing Technology R&D Center, Chengdu, Sichuan, 610207, China; 2 University of Electronic Science and Technology of China, Sichuan, 610054, China; 3 Sichuan New Material Research Center, Chengdu, 610207, Sichuan, China; 4 Institute of Chemical Materials, China Academy of Engineering Physics, Mianyang, 621900, Sichuan, China; 5 Beijing Computational Science Research Center, Beijing 100094, China

## Abstract

Systematic first-principles calculations were performed to investigate the adsorption and diffusion of Li on different graphene layers with B/N-doping and/or C-vacancy, so as to understand why doping heteroatoms in graphene anode could significantly improve the performance of lithium-ion batteries. We found that the formation of single or double carbon vacancies in graphene are critical for the adsorption of Li atoms. While the N-doping facilitates the formation of vacancies, it introduces over binding issue and hinders the Li diffusion. The presence of B takes the excessive electrons from Li and N and reduces the energy barrier of Li diffusion on substrates. We perceive that these clear insights are crucial for the further development of graphene based anode materials for lithium-ion batteries.

With excellent characters of high energy density, long cycling life and environmental friendliness, lithium-ion batteries (LIBs) have been widely used in portable electronic devices^1^,^2^,^3^,^4^,^5^. The performance of LIBs is mainly determined by the intrinsic properties of their electrodes. Therefore much attention has been paid in the recent years on exploring and developing novel electrode materials. Carbonaceous materials, such as graphite^6^,^7^, carbon nanofibers^8^,^9^, carbon nanotubes^10^,^11^,^12^ and porous carbon^13^,^14^, are promising anode materials in LIBs due to their high Li-storage capacity, high conductivity, decent electrochemical activity and low cost^15^,^16^. In particular, graphene has attracted extensive research interests with a theoretical maximum lithium capacity of 784 mAh/g by forming Li_2_C_6_ structure^17^, and an even higher capacity up to 1488 mAh/g for an isolated graphene flake that is only 0.7 nm in diameter^18^,^19^. However, it was found that the Li capacity of some graphene samples can be even significantly lower than that of bulk graphite^20^, possibly due to the formation of small Li clusters on graphene as the interaction between Li atoms^21^,^22^,^23^ is much stronger than that between Li and pristine graphene^24^,^25^,^26^.

Many approaches have been pursued to functionalize graphene as an anode material and doping graphene with nitrogen (N) and/or boron (B) atoms is one of the most effective ways. For example, Reddy *et al*. found that the Li capacity of N-doped graphene layers produced by chemical vapor deposition (CVD) technique is almost doubled compared to that of pristine graphene because of the appearance of a large number of surface defects^27^. Wu *et al*. prepared N- and B-doped graphene samples by using a mixed gas of NH_3_BCl_3_ and Ar, which show a high reversible Li-capacity of >1040 mAhg^−1^ at a low rate of 50 mA^−1^, meaning that they can be charged and discharged quickly^28^. The significant enhancement in the performance of N-/B-doped graphene motivates active theoretical studies to understand the physical mechanism behind^29^,^30^,^31^,^32^. Zhou *et al*. showed that the carbon vacancies (V_C_) would enhance the Li adsorption on graphene due to large charge transfer from Li to the nearest neighbor carbon atoms^24^. Yu used density functional theory (DFT) with a dispersion correction to investigate the joint effect of N-dopant and V_C_ on the electronic properties of graphene and its activity toward Li adsorption. His/her work confirmed that the N-decorated graphene with single and double vacancies could greatly improve the reversible lithium capacity^31^. Liu *et al*. found that the substitutional B atoms cause slight electron deficiency, which makes the adsorption of Li on the C_3_B monolayer easy^26^. Nevertheless, these pictures are not much beyond intuition and the synergy between effects of B,N-dopants and C-vacancy decorations on the improvement of Li storage capacity and conductivity remains vague, which hinders the development of graphene as anode materials in LIBs.

In the present work, we performed systematic density functional theory (DFT) calculations for the structures and energetics of different B/N/V_C_-decorated graphene geometries, as well as the Li adsorption and diffusion on them. We sorted out the roles of doping and vacancy decoration on the improvement of Li-adsorption on graphene, and identified several structures that have good adsorption energy, low diffusion barrier and easy preparation in experiments. Our theoretical results provide instructive guidelines for the further development of high performance C-based anode materials for LIBs.

## Results and Discussions

### The formation and Li-adsorption-performance of G_N_*x*_V_*y*_ substrates

We first studied the adsorption configurations and energies of one Li atom on the decorated graphene cells with *x* N-atoms and *y* V_C_, G_N_*x*_V_*y*_ (*x* = 0–4 and *y* = 0–2), corresponding to a N-concentration of ~1.4–5.6%, comparable with the experimental range for graphene samples synthesized via the thermal reaction between graphene oxide and NH_3_ at high temperature^33^. After structural optimization, we see that for *y* = 0, i.e., N-substituted graphene without V_C_, N atoms prefer to stay far away from each other to minimize the perturbation to the graphene π-electron bonds, as depicted in Table S1 in the supplementary material (SM). The C-N bond length is about 1.40–1.43 Å, very close to the C-C bond length in the pristine graphene. For *y* = 1 and 2, i.e., N-doped graphene with V_C_, the N dopants prefer to connect with two C atoms and form pyridine-like structure, which introduces a strongly localized donor states near the Fermi level^34^,^35^. The corresponding C-N bond length reduces to ~1.34 Å, deviating away from the lattice position of pristine graphene.

**Figure 1:**
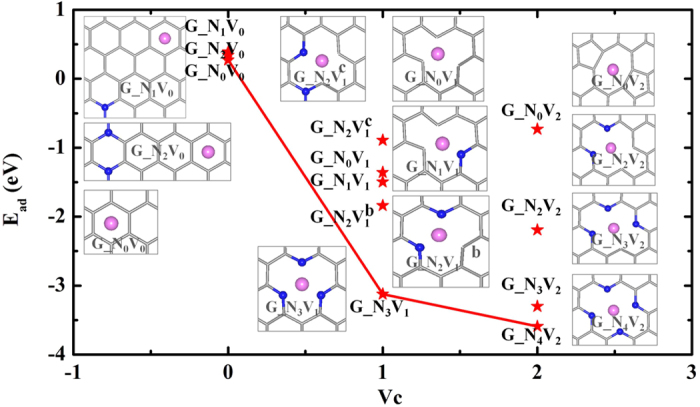
The adsorption energies (E_ad_) of one Li atom on the G_N_x_V_y_ graphene sheets as a dependence of carbon vacancies (V_C_). Insets are the corresponding atomic structures, where the gray sticks, blue and pink balls represent C, N and Li atoms, respectively.

For each system, we explored different Li adsorption sites (cf. Table S2 in SM) and those with the lowest adsorption energies, *E*_ad_, are shown in Fig. 1. We see that the *E*_ad_ of Li on the hollow site (H) of a 6 × 6 graphene supercell, 0.27 eV, is lower than that on the top site (T), 0.57 eV. The distance between Li and substrate, *d*_LG_, of the H-site, 1.72 Å, is also smaller than that of the T-site, 1.92 Å. These results indicate a single Li adatom prefers the hollow site on pristine graphene but Li atoms are more likely to form Li clusters since the value of *E*_ad_ is positive, in consistent with previous results^24^,^36^ (see details in Table S3 in SM). For the adsorption of one Li atom on the G_N_1_V_0_ and G_N_2_V_0_ systems, we found that Li prefers to stay on the H-site of graphene rather than any places near the N atoms. The *E*_ad_ of Li on G_N_1_V_0_ (G_N_2_V_0_) systems, 0.39 (0.34) eV, are also positive, and the corresponding values of *d*_LG_ are 1.75(1.76) Å, indicating that pure N-dopings in graphene don’t improve the lithium adsorption. Strikingly with the presence of V_C_ on pristine graphene, the *E*_ad_ decreases to negative values of −1.36 eV for single V_C_ and −0.73 eV for double V_C_s, as shown in Fig. 1, indicating that the adsorption of Li on the vacancy position is energetically preferred. The atomic positions in G_N_0_V_1_ have no obvious changes compared with perfect graphene due to the weak intraplanar relaxation^37^, whereas a typical 5–8–5 defect can be found in G_N_0_V_2_ with decreased C-C bond lengths near vacancies from 2.46 Å to 1.81 Å, as depicted in the insets in Fig. 1. *E*_ad_ values decrease further for Li on the N and V_C_-codoped graphene sheets. For example, *E*_ad_ becomes −3.12 eV for Li/G_N_3_V_1_, which implies a strong lithium adsorption on this substrate, as also demonstrated in previous experimental and theoretical studies^31^,^32^. We noted that the further increase of V_C_ number doesn’t significantly change the *E*_ad_ value, and the lowest *E*_ad_ is −3.59 eV, which occurs in Li/G_N_4_V_2_. The detailed analyses of adsorption structures show that the vertical *d*_LG_ is 1.43 Å for Li/G_N_3_V_1_, and decreases to 0.10 Å for Li/G_N_4_V_2_, implying that the Li atom is in the hole position surrounded by four pyridine N atoms and almost in the same plane with the N-doped graphene. Overall, the red curve in Fig. 1 that connects the lowest *E*_ad_ of Li on different graphene cells suggests that the carbon vacancies play the key role in the adsorption of Li on graphene.

**Figure 2:**
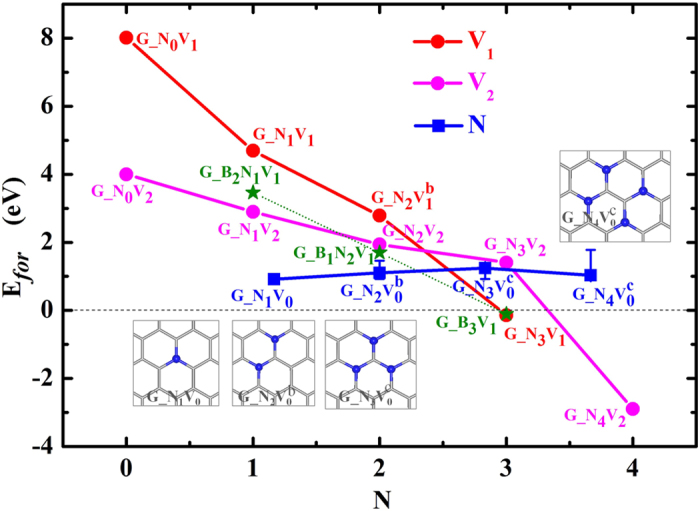
The formation energies (E_for_) of C-vacancies and N-dopings as a dependence of the number of nitrogen atoms in the G_N_x_V_y_ substrates. Insets show the G_N_x_V_0_ structures used in E_for_ calculations without C-vacancy, where the gray sticks and blue balls represent C and N atoms, respectively.

Now the question is how easy to create a C-vacancy on graphene? We note that the calculated formation energy of one V_C_ on graphene, *E*_*for*_(V_C_), shown in Fig. 2 is extremely high, 8.01 eV. In contrast, *E*_*for*_(V_C_) drops to 4.69 eV on N-doped graphene, and the trend of decrease continues with the further increase of N atoms, as shown in the red line in Fig. 2. The *E*_*for*_(V_C_) of G_N_3_V_1_ has a small negative value, indicating the easy formation of the N-V_C_ structure as a group. Similar trend appears for the formation of double-vacancies in graphene, with a very low *E*_*for*_(V_C_) of −2.89 eV for G_N_4_V_2_. The formation energy of single N-dopant on graphene, *E*_*for*_(N), is 0.91 eV, comparable with the previous DFT studies of 0.79 eV^38^ and 0.97 eV^31^. Interestingly, this value doesn’t change significantly with the increase of N dopants, as depicted in the blue line in Fig. 2 and the red curve in Figure S1(a). Here the blue solid squares show the *E*_*for*_(N) of single N-dopant on the G_N_*x*_V_0_ substrates (shown in the insets) that were used in *E*_*for*_ calculations of single C-vacancy, and the error bar illustrates the *E*_*for*_(N) window for different doping configurations of N on G_N_*x*_V_0_ (cf. more details in Table S1 in SM). Clearly, it is rather easy to insert N atoms in graphene, which serves as the precursor step for the formation of C-vacancies and consequently, enhances the Li adsorption. Considering the values of *E*_*for*_ of V_C_ and N-dopings and the values of *E*_*ad*_ of Li, we believe that G_N_3_V_1_ and G_N_4_V_2_ configurations can be easily formed and they are responsible to the enhancement of lithium capacity of graphene anode materials.

### Adsorption and diffusion of Li on B- and N-codoped G_B_*x*_N_3−*x*_V_1_ substrates

As we mentioned above, the graphite-like B/C/N layered materials prepared using CVD or other methods showed good performances as the anode matrix of LIBs^39^,^40^,^41^. Various precursors were used in experiments to create B dopings, including B_4_C, NaBH_4_, B_2_O_3_ and so on. So theoretically the *E*_*for*_ of single B-doping on graphene, *E*_*for*_(B), changes from −5.30 eV under B-rich conditions to +8.09 eV under oxidation conditions, as shown in Figure S1(b) in SM. Although the average values of *E*_*for*_(B) under typical experimental conditions, ranging from −0.23 eV with B_4_C precursor to +1.47 eV with NH_3_BH_3_ precursor, are a little higher than *E*_*for*_(N), the calculated *E*_*for*_(V_C_) of single C-vacancy on G_B_3_V_1_ is comparable with that of G_N_3_V_1_, and interestingly, its value for the B/N co-doped graphene, G_B_*x*_N_3-*x*_V_1_ (*x* = 0–3), is even lower than the corresponding value for the pure N-doped graphene by ~1.20 eV, as presented by the green stars in Fig. 2.

**Figure 3:**
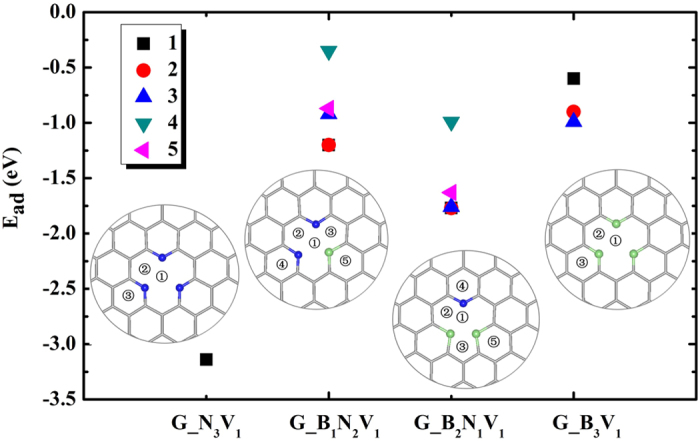
The adsorption energies of G_B_x_N_3-*x*_V_1_ systems and insets are the corresponding optimized atomic structures. The initial adsorption sites of Li are denoted as numbers. The gray sticks, green and blue balls represent C, B, and N atoms, respectively.

Then we give the values of *E*_ad_ for Li on several adsorption sites of G_B_*x*_N_3-*x*_V_1_ substrates, as depicted in Fig. 3. For Li on G_B_1_N_2_V_1_, we found that Li in the ground state is located at the vacancy site, close to the B dopant with a *d*_LG_ of 1.65 Å. A similar adsorption structure was obtained for Li/G_B_2_N_1_V_1_, but the Li atom is much closer to the N dopant instead of B. Strikingly, the most stable adsorption site of Li on G_B_3_V_1_ substrate is on the hollow site of the hexagonal ring adjacent to the B atom. Overall, the B and N co-doped graphene substrates give negative adsorption energies, and the values of *E*_*ad*_ of one Li atom on G_B_*x*_N_3−*x*_V_1_ are in the order: G_N_3_V_1_ (−3.12 eV) < G_B_2_N_1_V_1_ (−1.77 eV) < G_B_1_N_2_V_1_ (−1.20 eV) < G_B_3_V_1_ (−0.99 eV). Obviously, all of them are more active toward the adsorption of Li, compared with pristine graphene. Using G_B_2_N_1_V_0_ as an example, we also studied the *x*-dependence of *E*_*ad*_ (see Table S4 in SM) and found that *E*_*ad*_ decreases with the doping concentration of B till it is smaller than 2.78%.

**Figure 4:**
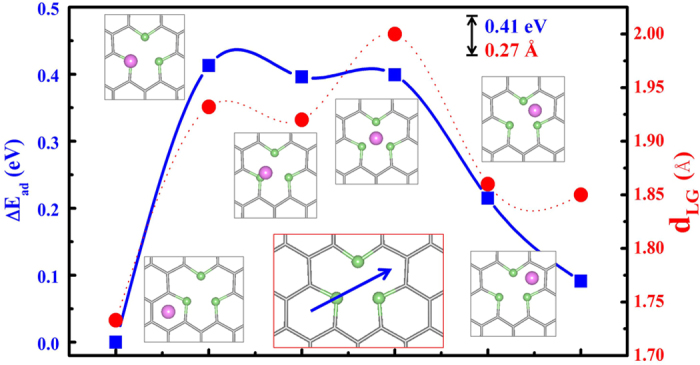
The relative adsorption energy (left axis) and the distance between Li and G_B_3_V_1_ substrate (right axis) at different adsorption sites. Insets are the corresponding adsorption configurations and the one with a red border shows the diffusion pathway.

While adsorption energies determine the Li capacity, the energy barrier of Li diffusion dictates the efficiency of charge and discharge cycles. Using the G_B_3_V_1_ substrate as an example, we calculated the *E*_ad_ of Li on selected adsorption sites along the diffusion pathway of H→T→V_C_→H, as shown in the inset in Fig. 4 highlighted by the blue arrow, and obtained the energy barrier of Li diffusion by using Δ*E*_ad_ = *E*_ad_(site) − *E*_ad_(H). For each adsorption site, we fixed the lateral coordinates of Li and optimized all the other atomic positions. The energy barrier for the movement of one Li atom on G_B_3_V_1_ is only 0.41 eV, and the largest difference in *d*_LG_ is about 0.27 Å. The low energy barrier and the small change in *d*_LG_ indicate a relatively flat potential energy surface of G_B_3_V_1_ for the Li movement. Similarly, we studied the diffusion of Li on G_B_2_N_1_V_1_ and obtained Δ*E*_ad_ of 0.80 eV and 0.48 eV along two different pathways, and the corresponding difference in *d*_LG_ of 0.83 Å and 0.56 Å, respectively (see Figure S2 in SM). Note that without B, the Δ*E*_ad_ of Li diffusion on G_N_3_V_1_ is 2.62 eV^32^, 6.5 times larger than that of G_B_3_V_1_. Clearly, the lithiation-delithiation process of Li on decorated graphene needs the joint effects of vacancy, B- and N-dopants.

### Electronic properties of decorated graphene substrates

**Figure 5:**
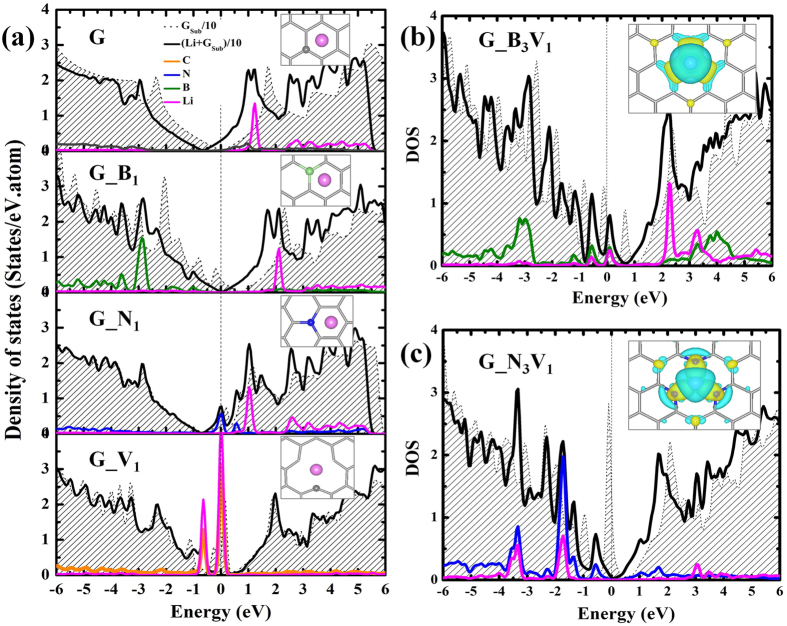
The total and partial density of states (DOS) of (**a**) G, G_B_1_, G_N_1_, and G_V_1_ substrates, (**b**) G_B_3_V_1_ and (**c**) G_N_3_V_1_ substrates. The solid line and dashed line with shadow in each panel denote the DOS of substrates with and without Li adsorption, respectively. Zero energy gives the position of the Fermi level. Insets in (**a**) show the local atomic positions, where the gray sticks, green, blue and pink balls represent C, B, N and Li atoms, respectively. Insets in (**b**) and (**c**) are the electron redistributions within the range of ±2 × 10^−3^ e/Å^3^, and the yellow and blue isosurfaces represent electron accumulations and depletions.

We performed detailed analyses on the electronic properties of various graphene substrates with/without Li adsorption, so as to understand the role of doping and/or vacancy on the Li adsorption. The total and partial density of states (DOS) of pristine graphene (G), G_B_1_, G_N_1_, and G_V_1_ are plotted in Fig. 5(a). The dashed line with shadow in the uppermost panel in Fig. 5(a) shows a perfect Dirac state of the pristine graphene at the Fermi level (*E*_*f*_). The adsorption of Li atom keeps the DOS profile almost unchanged, but the *E*_*f*_ moves to the conduction states due to the addition of 1*e* from Li. As was well known, the *E*_*f*_ of B(N)-doped graphene is shifted to the valance(conduction) band, as shown in the middle panels in Fig. 5(a), indicating that G_B_1_(G_N_1_) is a hole (electron)-rich material. The adsorption of Li on G_B_1_ substrate compensates the hole attraction and the Dirac state of Li/G_B_1_ moves back to *E*_*f*_, making the system stable with a negative *E*_ad_ of −1.4 eV^32^. In contrast for Li/G_N_1_, we see an obvious peak existing on the *E*_*f*_, coming from the N-*p*_*z*_ orbital. For these three systems, there is little hybridization between Li and substrate/dopant. For example, the B-*p*_*x,y*_ states in G_B_1_ locate at ~−2.9 eV below the *E*_*f*_, but the Li-*s* and -*p*_*z*_ states appear at ~2.1 eV above the *E*_*f*_. The attractive/repulsive electrostatic interactions between Li and substrates lead to a negative/positive *E*_ad_ values. Strikingly, for G_V_1_ substrate, as shown in the lower panel in Fig. 5(a), there is a big peak around *E*_*f*_ caused by the hybridization of Li-*p*_*x,y*_ and C-*p*_*x,y*_ in-plane orbitals, and the adjacent peak at −0.7 eV below the *E*_*f*_ is composed of the Li-*s* and C-*p*_*x,y*_ orbitals. Thus the interaction between Li and G_V_1_ substrate is very strong, leading to a low *E*_ad_ value.

Interestingly, we found that the DOS profile of G_B_3_V_1_ substrate in Fig. 5(b) is very similar with that of G_B_1_, with peaks of B-*p*_z_ orbital near the *E*_*f*_. The adsorption of Li makes the B-*p*_z_ states shift to lower energies, but the hybridization of Li-*s,p* and B-*p* states is still relatively weak near the *E*_*f*_. Similar with G_V_1_, the DOS of G_N_3_V_1_ also has a big peak right at the *E*_*f*_, coming from the dangling bonds at the N atom sites. The orbital hybridizations of N-*p* and Li-*s*,*p* orbitals can be observed around −1.8 eV and −3.5 eV, which are responsible for the lower *E*_ad_ of G_N_3_V_1_ than G_B_3_V_1_. The interaction between Li and substrate can be described more clearly by the electron redistribution, *n(**r***) = *n*_Li/sub_(***r***) − *n*_sub_(***r***) − *n*_Li_(***r***), obtained from electron densities of Li on substrates, pure substrates and Li atom. As displayed in the insets in Fig. 5(b) and (c), there are obvious electron transfer from Li to dopants which enhances the binding of Li on decorated graphene substrates. The contrast between G_B_3_V_1_ and G_N_3_V_1_ manifest through the influence of N extending to the second nearest neighbors of C atoms due to the symmetry breaking of the π-electron system, whereas B appears to only affect the nearest neighbor C atoms.

In summary, we performed systematic first-principles calculations to study the adsorption and diffusion of Li on decorated graphene with B and/or N dopants and C vacancies. Our results indicate that V_C_ sites (<3% in this study) rather than the dopants serve as attractive centers for Li adsorption on graphene. While the formation energy of one V_C_ on graphene is as high as 8.01 eV, the N-doping drastically decreases this value to as low as −0.14 eV for G_N_3_V_1_. The co-doping of B keeps negative adsorption energies for Li, and significantly reduces the energy barrier of Li diffusion, e.g., 0.41 eV for Li/G_B_3_V_1_ and 0.48 eV for Li/G_B_2_N_1_V_1_. The electronic structure analyses show that the interaction between Li and B states is rather weak, whereas Li has a strong orbital hybridization with N-*p* states, causing a high diffusion barrier. Our theoretical studies provide clear insights for the understanding of the individual roles of doping and vacancy decorations for the performance of enhancement of N(B)-doped graphene as electrode materials in LIBs, and provide guidelines for the design of new battery materials.

## Methods

Our density functional theory calculations were performed using the Vienna Ab initio Simulation Package (VASP)^42^,^43^. Projector-augmented-wave (PAW) potential and the PW91 version of general gradient approximation (GGA)^44^ were employed to describe the electron-ionic core interactions and the exchange-correlation interaction among electrons, respectively. Our preliminary calculations by using the vdW-DF2 version of nonlocal van der Waals functional^45^,^46^ show that the inclusion of dispersion corrections in DFT don’t change the main features of Li adsorption on decorated graphene. We used an energy cutoff of 500 eV for the plane-wave basis expansion and a size-dependent Monkhorst-Pack *k*-points sampling in the Brillouin zone (BZ). The crystal constant and positions of the ions were fully relaxed until the final force on each atom is smaller than 0.01 eV/Å. The optimized in-plane lattice constant of graphene primitive cell is 2.47 Å, in good agreement with the experimental values of 2.46 Å derived from the X-ray crystallography of AB graphite and with previous theoretical predictions^36^. For the adsorption and diffusion of one Li atom on decorated graphene, we used a periodic slab consisting of a 6 × 6 graphene supercell and a vacuum layer of 12 Å along the normal direction to avoid the interaction between two adjacent images.

To assess the stability of Li adsorbed on graphene, we calculate the adsorption energy (*E*_ad_) of a Li adatom as follows


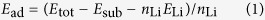


where *E*_tot_ and *E*_sub_ are the total energies of the graphene after and before lithium adsorption, respectively, *n*_Li_ is the number of Li adatoms, and *E*_Li_ is the energy of one Li atom in the body-centered cubic crystal phase. A negative value of *E*_ad_ implies a spontaneous adsorption of Li atoms.

The formation energies (*E*_*for*_) of defects, including N-doping and C-vacancy, on graphene are calculated as follows:


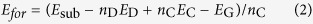


where *E*_sub_ and *E*_G_ are the total energies of decorated graphene and pristine graphene, respectively; *E*_D_ and *E*_C_ are the energies of one N atom in N_2_ gas and one C atom in graphene, respectively; and *n*_D_ and *n*_C_ are the numbers of N-dopants and C-vacancies in the supercell, respectively.

## Additional Information

**How to cite this article**: Jin, M. *et al*. Enhanced Absorption and Diffusion Properties of Lithium on B,N,VC-decorated Graphene. *Sci. Rep.* **6**, 37911; doi: 10.1038/srep37911 (2016).

**Publisher's note:** Springer Nature remains neutral with regard to jurisdictional claims in published maps and institutional affiliations.

## Supplementary Material

Supplementary Material
