# Supplementary material for: Enhanced Absorption and Diffusion Properties of Lithium on B,N,VC-decorated Graphene
Source: Sci Rep. 2016 Nov 29;6:37911. doi: 10.1038/srep37911 (PMC5126578; doi:10.1038/srep37911)
Supplement: Supplementary Material [file srep37911-s1.doc]

**Enhanced Absorption and Diffusion Properties of Lithium on B,N,V_C_-decorated Graphene**

Mengting Jin^1^, L.C. Yu*^3^*, W.M. Shi^4,5^, J.G. Deng^4,5^, Y.N. Zhang^1,2^*^*^*

*^1^Chengdu Green Energy and Green Manufacturing Technology R&D Center, Chengdu, Sichuan, 610207, China*

*^2^Beijing Computational Science Research Center, Beijing 100094, China*

*^3^University of Electronic Science and Technology of China, Sichuan, 610054, China*

*^4^Sichuan New Material Research Center, Chengdu, 610207, Sichuan, China*

*^5^Institute of Chemical Materials, China Academy of Engineering Physics, Mianyang, 621900, Sichuan, China*

***^*^****Corresponding author:* [*yanningz@csrc.ac.cn*](mailto:yanningz@csrc.ac.cn)

**Table S1.** The calculated formation energy (E*_f_*) of N-substituted graphene without any carbon vacancies


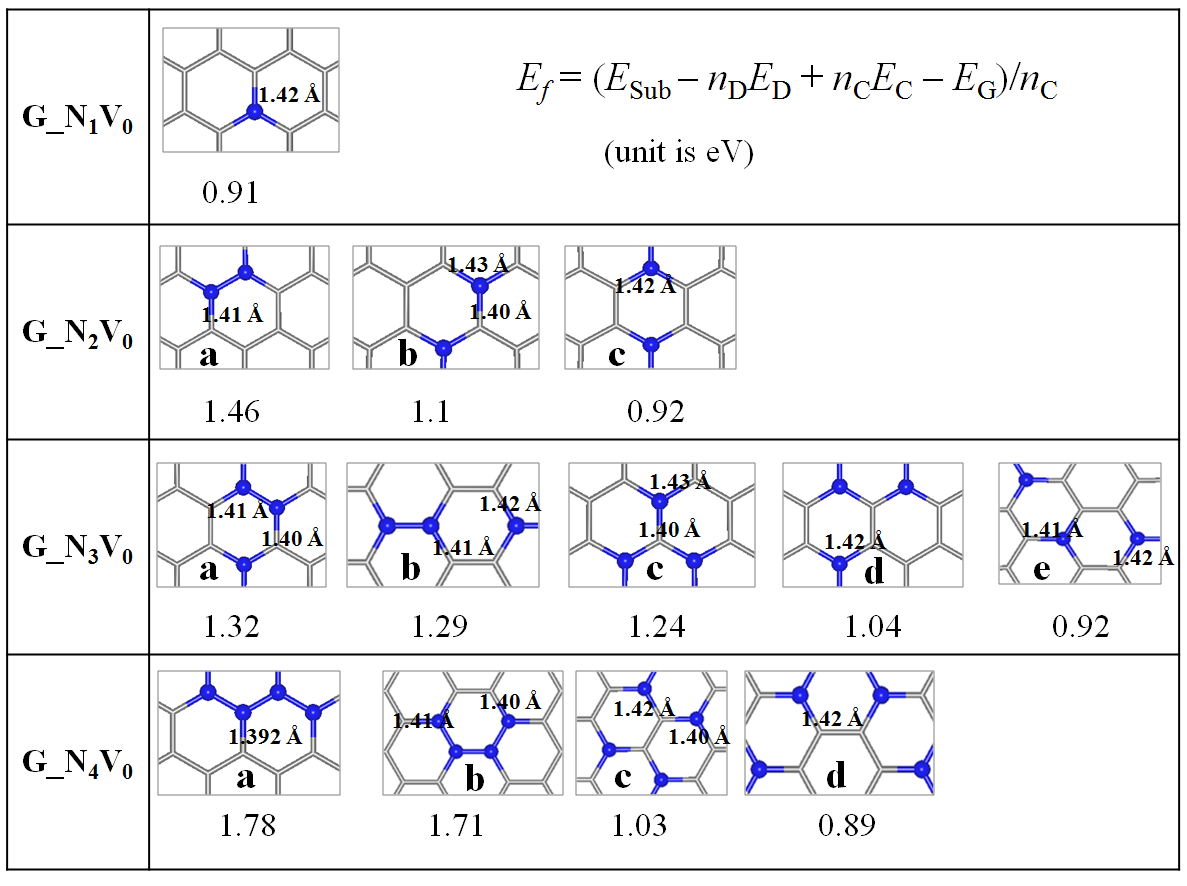


**Table S2.** The adsorption energies (*E_ad_*) of one Li atom on the G_N*_x_*V*_y_* graphene sheets with different adsorption sites


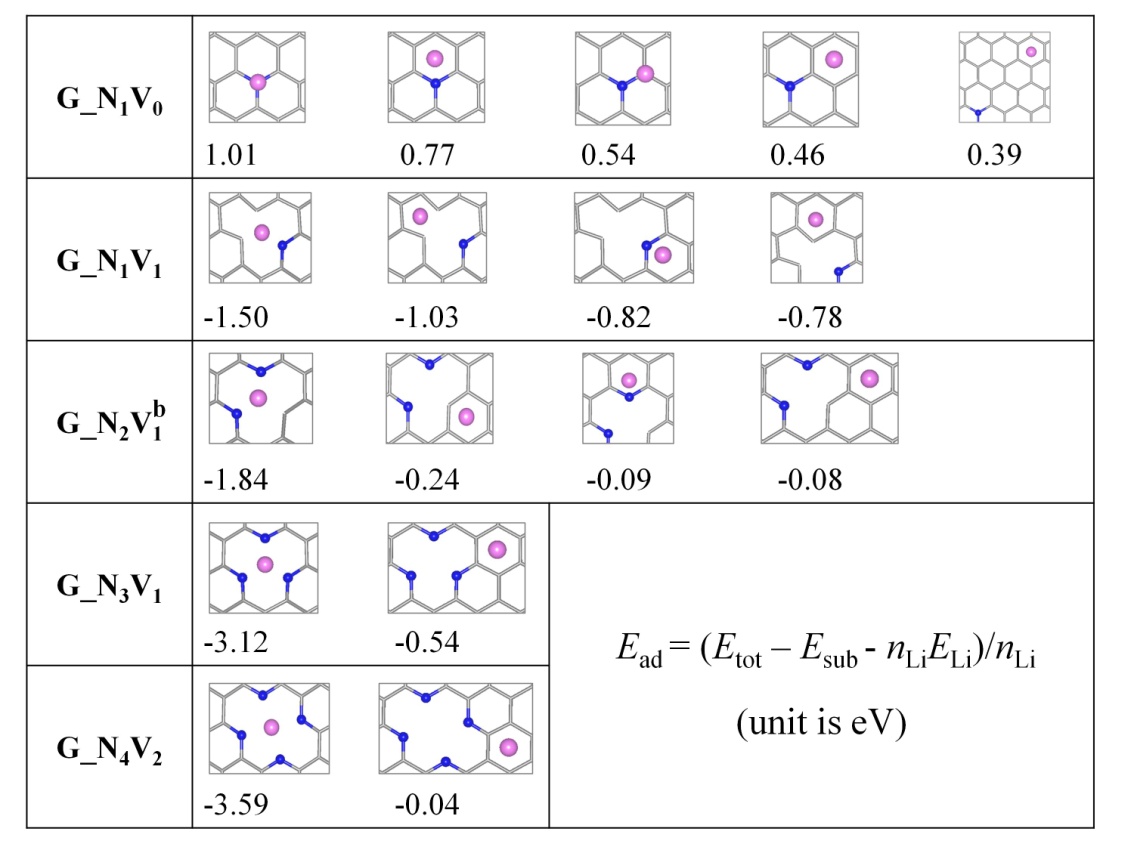


**Table S3** Calculated adsorption energy (E_ad_) and distance between the lithium atom and the graphene layer (d_LG_) of the lithium adsorption on pristine graphene

|  | **supercell** | | **d_LG_ of Top(Å)** | **E_ad_ of Top(eV)** | **d_LG_ of Hollow(Å)** | **E_ad_ of Hollow(eV)** |
| --- | --- | --- | --- | --- | --- | --- |
| This work | 6×6 | 1.92 | | 0.57(-1.04) | 1.72 | 0.27(-1.33) |
| *Ref ^a^* | 6×6 |  | | 0.62 (-0.98) |  | 0.32(-1.29) |
| *Ref ^b^* | 6×6 | 2.01 | | (-1.15) | 1.71 | (-1.58) |
| *Ref ^c^* | 4×4 | 1.89 | | (-0.75) | 1.71 | (-1.10) |
| *Ref ^d^* | 4×4 |  | |  | 1.84 | (-1.21) |

*a*. J. Phys. Chem. C 116, 21780 (2012),

*b*. J. Chem. Theory Comput. 8, 1064−1071(2012)

*c*. Phys. Rev. B 77, 235430 (2008)

*d*. Phys. Rev. B 75, 075401(2007)


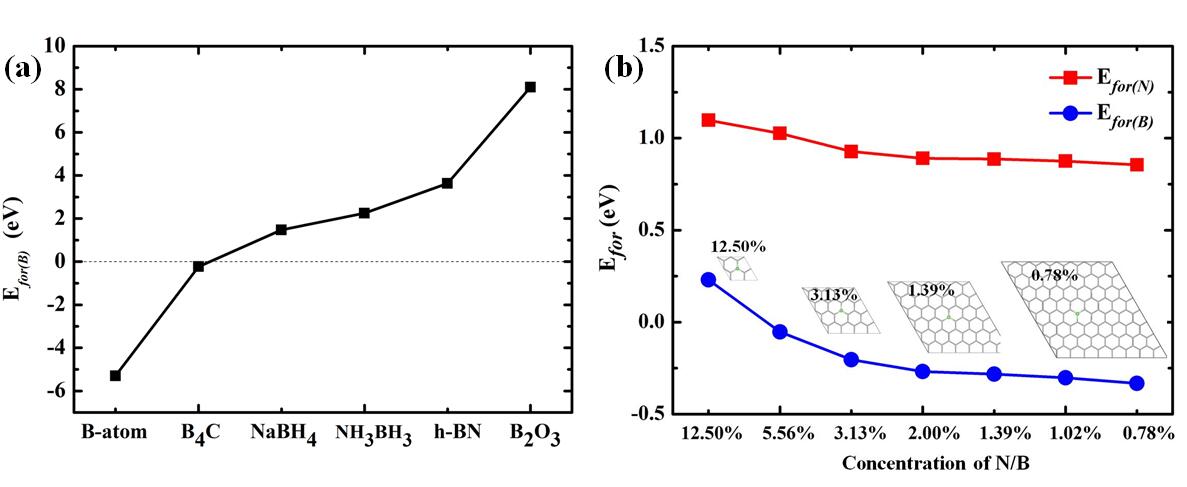


**Figure S1** (a) The *E_for_* of single B-doping on graphene in various precursors. (b) The E*_for_* of single N (single B) in graphene changed with its content. Here, the energies of one N atom in N_2_ gas and one B atom in B_4_C were adopted as energy references.

**Table S4** Adsorption energies (E_ad_) of Li and concentration of B in G_B_2_N_1_V_1_ with different supercell sizes

| **Supercell size** | **E_ad_ (eV)** | **Concentration of B** |
| --- | --- | --- |
| 3×3 （18 atoms） | -1.36 | 11.1% |
| 4×4 （32 atoms） | -1.50 | 6.25% |
| 5×5 （50 atoms） | -1.58 | 4% |
| 6 ×6 （72 atoms） | -1.77 | 2.78% |
| 7 ×7 （98 atoms） | -1.76 | 2.04% |
| 8 ×8 （128 atoms） | -1.76 | 1.56% |


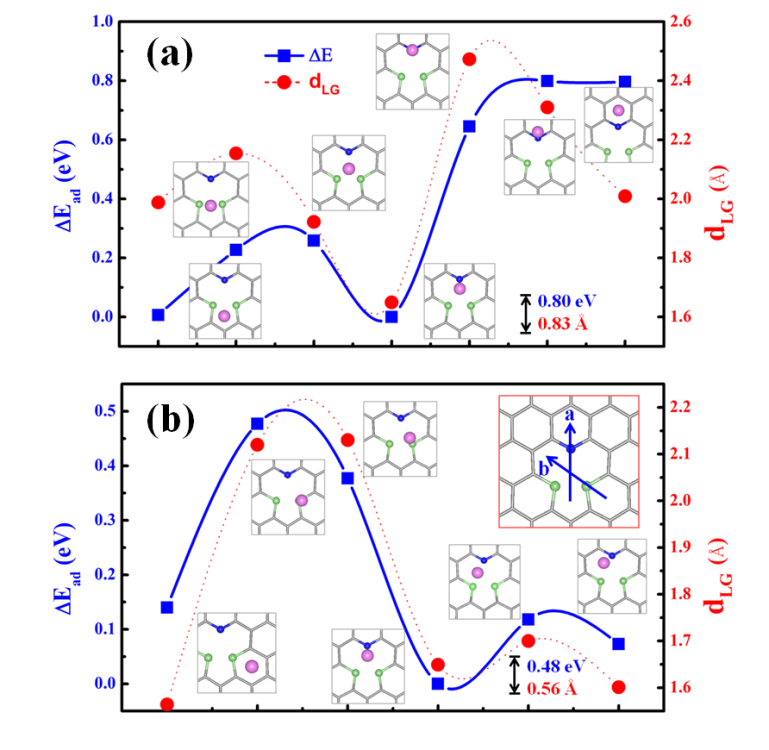


**Figure S2** The relative adsorption energy (left axis) and the distance between Li and G_B_2_N_1_V_1_ substrate (right axis) at different adsorption sites. Insets are the corresponding adsorption configurations and the one with a red border shows the two diffusion pathways.
